# Response Rates and Transplantation Impact in Patients with Relapsed Acute Promyelocytic Leukemia

**DOI:** 10.3390/cancers16183214

**Published:** 2024-09-21

**Authors:** Alessandro Costa, Carmelo Gurnari, Emilia Scalzulli, Laura Cicconi, Luca Guarnera, Ida Carmosino, Raffaella Cerretti, Maria Laura Bisegna, Saveria Capria, Clara Minotti, Anna Paola Iori, Lorenzo Torrieri, Adriano Venditti, Alessandro Pulsoni, Maurizio Martelli, Maria Teresa Voso, Massimo Breccia

**Affiliations:** 1 Hematology Unit, Businco Hospital, Department of Medical Sciences and Public Health, University of Cagliari, 09121 Cagliari, Italy; alessandrocosta161195@gmail.com; 2 Department of Biomedicine and Prevention, University of Rome Tor Vergata, 00133 Rome, Italy; 3 Hematology, Department of Translational and Precision Medicine, Az. Policlinico Umberto I-Sapienza University, 00161 Rome, Italy; 4 Department of Hematology, Polo Universitario Pontino, S.M. Goretti Hospital, 04100 Latina, Italy

**Keywords:** acute promyelocytic leukemia, disease relapse, arsenic trioxide, all-*trans* retinoic acid, allogeneic hematopoietic cell transplant

## Abstract

**Simple Summary:**

Relapses of acute promyelocytic leukemia (APL) remain a challenge despite the introduction of all-*trans* retinoic acid (ATRA) and arsenic trioxide (ATO). Given the variability in salvage therapies and emerging evidence supporting the deferral of hematopoietic cell transplantation (HCT) in patients receiving ATO, we analyzed the outcomes of a multicentric cohort of 67 relapsed APL patients. Better outcomes were reported with ATO ± ATRA compared to chemo-based regimens and ATRA ± Gemtuzumab ozogamicin (GO). A significant survival advantage was observed for patients undergoing HCT in the chemo-based cohort (*p* = 0.017), but not in the ATO-based group (*p* = 0.12). Achieving molecular complete remission (CR) post salvage therapy emerged as the main prognostic factor for second relapses in both univariate and multivariate analyses. Our findings support the efficacy of ATO-based therapies in first relapse and enhance the role of molecular remission as an independent outcome predictor in both first and second APL relapses.

**Abstract:**

Background: The introduction of all-*trans* retinoic acid (ATRA) and arsenic trioxide (ATO) has radically improved the prognosis of acute promyelocytic leukemia (APL), with cure rates above 80%. While relapse occurs in less than 20% of cases, addressing this issue remains challenging. Identifying effective salvage therapies for relapsed APL is crucial to improve patient outcomes. Methods: A retrospective analysis was performed on a multicentric cohort of 67 APL patients in first relapse, treated in three Italian hematology centers from June 1981 to November 2021. The overall survival (OS) and cumulative incidence of relapse (CIR) were calculated, and predictive factors were assessed using Cox regression models. Results: Overall, 61 patients (91%) received ATO ± ATRA (40.3%), chemo-based regimens (40.3%), or ATRA ± Gemtuzumab ozogamicin (GO) (10.4%). Complete remission (CR) was achieved in 98.2% of patients (molecular CR, n = 71.4%). With a median follow-up time of 54.5 months, the 5-year OS was 73% in the ATO ± ATRA group, 44% in the chemo-based group, and 29% in the ATRA ± GO group (*p* = 0.035). The 5-year OS rate was also higher for transplant recipients vs. non-recipients within the chemo-based cohort (50% vs. 33%, *p* = 0.017), but not in the ATO-based cohort (*p* = 0.12). ATO-based salvage therapy resulted in better OS in both univariate (*p* = 0.025) and multivariate analyses (*p* = 0.026). The 2-year CIR was higher in patients without molecular CR vs. patients in molecular CR (66% vs. 24%, *p* = 0.034). Molecular CR was a significant predictor of second relapse in both univariate (*p* = 0.035) and multivariate analyses (*p* = 0.036). Conclusions: Our findings support the efficacy of ATO-based therapies in first relapse of APL and confirm the achievement of molecular remission as an independent outcome predictor in both first and second APL relapse.

## 1. Introduction

Acute promyelocytic leukemia (APL) is a rare subtype of acute myeloid leukemia (AML) with distinct biological, clinical, and prognostic features [1]. The hallmark of APL is the balanced reciprocal translocation t (15;17) (q24.1;q21.1), resulting in the fusion of the promyelocytic leukemia (*PML*) gene on chromosome 15 with the retinoic acid receptor α (*RARα*) gene on chromosome 17, leading to the *PML::RARA* fusion gene [2]. The first revolutionary step in APL management was the introduction of all-*trans* retinoic acid (ATRA) in the 1980s, with long-term cure rates exceeding 80% [3]. Another milestone was the introduction of arsenic trioxide (ATO) in the late 1990s, which, in combination with ATRA, now constitutes the standard first-line treatment for standard-risk (<10 × 10^9^/L leukocytes) APL patients [4,5].

Despite significant advancements transforming APL from a rapidly fatal disease to the most curable subtype of AML, 10–20% of patients treated with ATRA and chemotherapy [3,6], and 1–5% of patients treated with ATO-ATRA, still experience relapse [4,5,7]. Although evidence is limited, the 2019 European LeukemiaNet (ELN) guidelines recommend ATO-ATRA as salvage therapy for patients relapsing after ATRA plus chemotherapy; conversely, patients relapsing after ATO-ATRA should receive ATRA plus chemotherapy [8]. Recommendations for the management of APL relapse are summarized in Table 1.

While there is consistent evidence supporting the efficacy of ATO in relapsed APL patients, the benefit of adding ATRA in relapsed disease is less clear. For instance, a study published in 2003 showed no benefit from adding ATRA to ATO in this setting [9]. However, two more recent studies reported a 100% second complete response (CR) rate in two cohorts of 31 and 22 relapsed APL patients treated with ATO-ATRA, with molecular CR rates of 80% and 91%, respectively [10,11]. Further evidence supports ATO-based salvage therapy even in patients who previously received ATO-based first-line therapy. For instance, among 67 APL patients firstly administered with ATO-based therapy and salvaged with an ATO-based reinduction regimen at relapse, 94% achieved a second molecular CR [12].

Although randomized controlled trials are unfeasible due to the rarity of the events, autologous hematopoietic cell transplantation (HCT) is the preferred option for patients with relapsed APL in second molecular CR, while allogeneic HCT is recommended for patients with molecular persistence and subsequent relapses [9]. In this context, ATO has demonstrated the ability to increase the rate of molecular CR, reducing post-autologous HCT relapses [13]. Additionally, recent evidence suggests the possibility of deferring transplantation in patients in second CR who receive salvage therapy with ATO [14]. Given the significant variability in salvage therapies for relapsed APL and recent data suggesting the deferral of transplantation in those achieving a second CR when treated with ATO-based regimens, we leveraged our regional network to analyze our experience in this setting.

## 2. Materials and Methods

We performed a retrospective analysis of 67 patients with APL in first relapse, aged ≥18 years, who were followed at three major Italian hematology centers of the Lazio region from June 1981 to November 2021. Informed consent was obtained from treated patients as per institutional protocols.

Definition of relapse and response criteria were based on the revised International Working Group criteria [15]. Morphological CR was defined as the restoration of normal marrow cellularity with a promyelocytes count below 5%, a peripheral polymorphonuclear leukocyte count of >1.5 × 10^9^/L, and a platelet count of >100 × 10^9^/L. Molecular CR was defined as the achievement of negative results at quantitative *PML::RARα* transcript analyses by real-time PCR (RT-PCR). Morphological relapse was defined as the loss of CR, and molecular relapse was defined as the reappearance of positive RT-PCR results at any time after achieving post-consolidation molecular CR, confirmed in two bone marrow samples taken in close temporal proximity, as per established guidelines [8].

Continuous variables were reported as medians with interquartile ranges (IQR), and categorical variables were presented as frequencies and percentages. Group comparisons were performed using chi-square tests, Fisher’s exact test, ANOVA test for continuous variables, and the Wilcoxon-Mann-Whitney test for non-parametric series comparison. The overall survival (OS) was calculated from the date of relapse to death or last follow-up. The cumulative incidence of relapse (CIR) was defined as the time from first to second disease relapse in a competitive setting, with death from other causes as event, and the Gray’s test was used for CIR comparisons. Survival analysis was performed using Kaplan–Meier curves, with differences assessed by the Log-Rank test. Univariate and multivariate analyses were conducted using Cox proportional hazards regression to determine hazard ratios (HR) and 95% confidence intervals (95% CI) for factors associated with survival. Statistical analysis was performed with IBM Corp Released 2023. IBM SPSS Statistics for Macintosh, Version 29.0.2.0 Armonk, NY: IBM Corp., and with R Core Team (2021). R: A Language and Environment for Statistical Computing. R Foundation for Statistical Computing, Vienna, Austria. A level of *p* < 0.05 was used to define statistical significance in all tests.

## 3. Results

### 3.1. Study Population

Table 2 summarizes patient characteristics, both at baseline and at relapse. The median age at relapse was 48.2 years (IQR: 34.9–71.3), with a male-to-female ratio of 1.16. First line regimen included the Gruppo Italiano Malattie EMatologiche dell’Adulto (GIMEMA) AIDA0493 protocol [16] in 23 patients (34.3%), AIDA2000 [1] in 38 patients (56.7%), and the APL0406 regimen [5] in three patients (4.5%), while three patients received a combination of AIDA + Gemtuzumab ozogamicin (GO), idarubicin (IDA) + cytarabine (Ara-C), and daunorubicin (DNR), respectively. Overall, the median follow-up time was 54.5 months (IQR: 11.7–126.4), and the median time from APL diagnosis to relapse was 17.3 months (IQR: 12.2–35.3). Morphological relapse occurred in 62.7%, while 25 patients (37.3%) experienced molecular relapse without morphological evidence of leukemic blasts. Additionally, 29 patients (58.0%) relapsed during first-line maintenance therapy. Of them, 12 patients (41.3%) were at high risk according to Sanz criteria at the time of diagnosis. Extramedullary relapses were reported in 11 patients (16.4%), mostly localized in the central nervous system (CNS) (81.9%).

### 3.2. Patient Characteristics and Distribution by Salvage Therapy and Transplant Consolidation

Patient characteristics and outcomes according to salvage therapy are summarized in Table 3. Overall, 61 patients (91.0%) received salvage therapy, while five patients died before having the opportunity to start treatment, and one patient with an extramedullary relapse received involved-site radiotherapy (ISRT). Specifically, 27 patients (40.3%) were given ATO ± ATRA, 27 patients (40.3%) were given chemo-based therapy [Ara-C and mitoxantrone (MITO), n = 22; IDA and Ara-C, n = 5], and seven patients (10.4%) were treated with ATRA ± GO.

No significant sex differences were observed across the three treatment groups (*p* = 0.481). However, patients receiving ATRA ± GO were older compared to those receiving other regimens (median age: 40.5 years vs. 31.0 years; *p* = 0.013). Additionally, there was a significant difference in relapse risk classes among the three groups (*p* = 0.010). Significant differences were also observed in first-line regimens (*p* < 0.001): AIDA2000 was used as first-line treatment for 92.6% of patients receiving ATO ± ATRA and 42.8% of those receiving ATRA ± GO, whereas AIDA0493 was the predominant first-line regimen (62.9%) for patients receiving chemo-based salvage therapy. 

After a median of 6.27 months (IQR: 4.0–8.0) from first relapse, 26 patients (42.6%) underwent consolidation with HCT, including 17 patients (25.4%) who received autologous HCT and 9 patients (13.4%) who received allogeneic HCT. The year of relapse for patients who underwent allogeneic HCT ranged from 1996 to 2007, while for those who received autologous HCT, it ranged from 1985 to 2011. Of them, four patients (44.4%) undergoing allogeneic HCT and two patients (11.8%) undergoing autologous HCT had extramedullary involvement at first relapse (*p* = 0.059). Patients undergoing autologous HCT were also significantly younger than those receiving allogenic HCT (33.4 years vs. 50.5 years, *p* < 0.001). Additionally, a higher proportion of patients undergoing autologous HCT were male (64.7%) compared to those receiving allogeneic HCT (22.2%) (*p* = 0.016). Among patients undergoing autologous HCT, 51.8% received chemo-based treatment, whereas 28.6% of those undergoing allogeneic HCT received ATRA ± GO (*p* = 0.565). Considering the 35 patients who did not undergo transplant-consolidation, 22 patients (62.8%) received ATO ± ATRA salvage therapy, nine patients (25.7%) received chemo-based regimens, and four (11.5%) patients received ATRA ± GO. After a median follow-up of 41.8 months (IQR: 10.3–96.7), the 5-year OS was 67% (95% CI, 49–90) in the ATO ± ATRA group, 33% (95% CI, 13–84) in the chemo-based group, and 25% (95% CI, 4.6–90) in the ATRA ± GO group (*p* < 0.001).

*Summary*: Non-transplanted patients treated with ATO ± ATRA as salvage therapy demonstrated superior 5-year OS compared to those on chemo-based regimens or ATRA ± GO.

### 3.3. Response Rates of Salvage Therapies, Transplant Outcomes, and Predictive Factors for Survival

**Figure 1:**
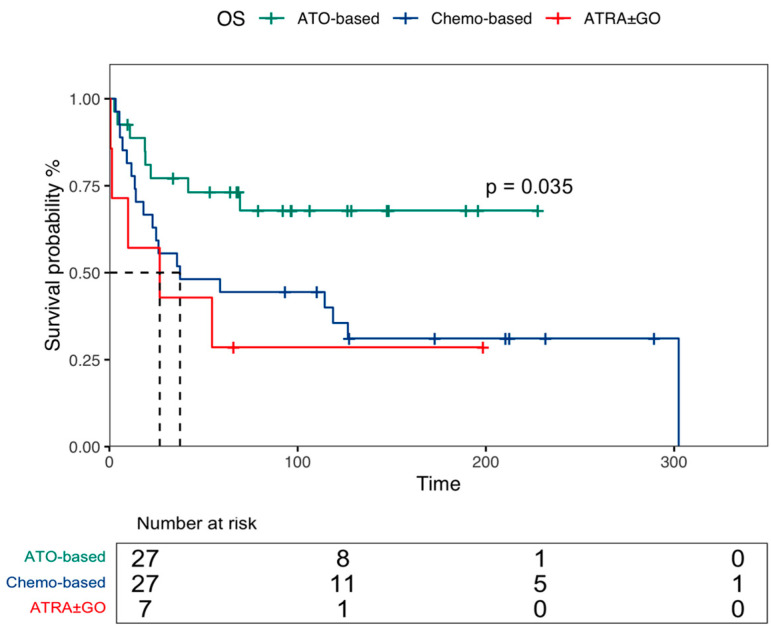
Overall survival (OS) according to salvage therapy received in relapsed patients with acute promyelocytic leukemia (APL).

Of the 56 evaluable patients, 55 (98.2%) achieved CR following salvage therapy. Specifically, molecular CR was attained in 40 patients (71.4%), while 15 patients (26.7%) achieved morphological CR with persistent molecular transcript. One patient (1.7%) had refractory disease and subsequently died due to disease progression. There were no statistically significant differences among the salvage therapy regimens in achieving either morphological or molecular CR (*p* = 0.112). Overall, the median OS was 114.3 months (95% CI, 33.8–194.8). Patients who relapsed during first-line maintenance had a significantly shorter median OS of 22.9 months (95% CI, 12.4–33.4) compared to those who either did not receive maintenance or had completed it, with a median OS of 126.6 months (95% CI, 63.3–189.6) (*p* = 0.045). No significant differences in OS were observed based on gender (*p* = 0.27) or age (<65 vs. ≥65 years, *p* = 0.16). As shown in Figure 1, after a median follow-up time of 54.5 months (IQR: 11.7–126.4) from relapse, the 5-year OS was 73% (95% CI, 58–92) in the ATO ± ATRA group. In contrast, it was 44% (95% CI, 29–68) for patients receiving a chemo-based regimen and 29% (95% CI, 8.9–92) for those treated with ATRA ± GO (*p* = 0.035).

**Figure 2:**
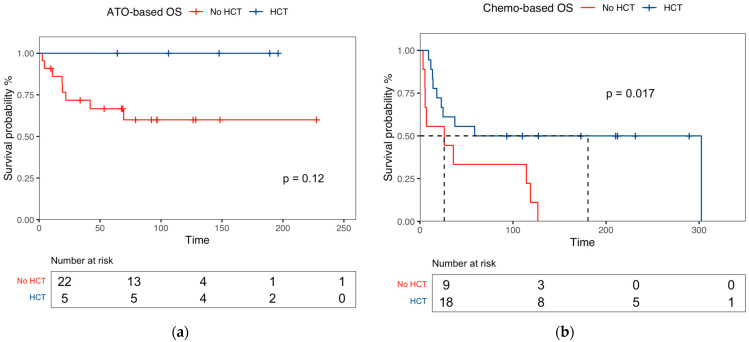
Overall survival (OS) according to transplant consolidation in (**a**) ATO-based and chemo-based (**b**) salvage therapies.

Moreover, after a median follow-up time of 57.9 months (IQR: 14.5–122.4), the 5-year OS was 59% (95% CI, 40–88) for autologous HCT, 56% (95% CI, 40–88) for allogenic HCT, and 51% (95% CI, 31–97) for non-transplanted patients (*p* = 0.33). Among patients receiving ATO ± ATRA, no statistically significant differences in the 5-year OS rates were observed between transplant recipients and non-recipients [100% vs. 67% (95% CI, 49–90), *p* = 0.12] (Figure 2a). Conversely, in the chemo-based salvage regimen cohort, there was a statistically significant difference in the 5-year OS between those who received consolidation with HCT and those who did not [50% (95% CI, 32–79) vs. 33% (95% CI, 13–84), *p* = 0.017] (Figure 2b).

In univariate analysis (Table 4), the time to first relapse from APL diagnosis [HR 0.96 (95% CI, 0.94–0.99), *p* = 0.016], the relapse during first-line maintenance [HR 1.95 (95% CI, 1.0–3.8), *p* = 0.049], and salvage therapy with ATO [HR 0.39 (95% CI, 0.1–0.8), *p* = 0.025] were significant predictors of OS. In multivariate analysis, relapse during first-line maintenance remained a significant predictor of adverse outcomes [HR 2.012 (95% CI, 1.1–3.8), *p* = 0.049], while ATO-based salvage therapy continued to be a significant independent predictor of favorable outcomes [HR 0.423 (95% CI, 0.1–0.89), *p* = 0.026].

*Summary*: No differences in second response rates were found across treatments. However, ATO ± ATRA led to superior 5-year OS compared to chemo-based regimens and ATRA ± GO. HCT positively impacted OS only in chemo-based regimens, with relapse during first-line maintenance and ATO-based therapy being key survival predictors.

### 3.4. Second Relapse Rates and Predictive Factors for Relapse

Overall, 26 patients experienced a second disease relapse, with a median time of 14.3 months (IQR: 10.7–27.5) from first relapse. After a median follow-up time of 26.0 months (IQR: 9.6–302.4), the 2-year CIR in the whole cohort was 34% (95% CI, 21–46), with no significant differences based on gender (*p* = 0.7), relapse during maintenance (*p* = 0.4), or use of HCT as consolidation (*p* = 0.5).

**Figure 3:**
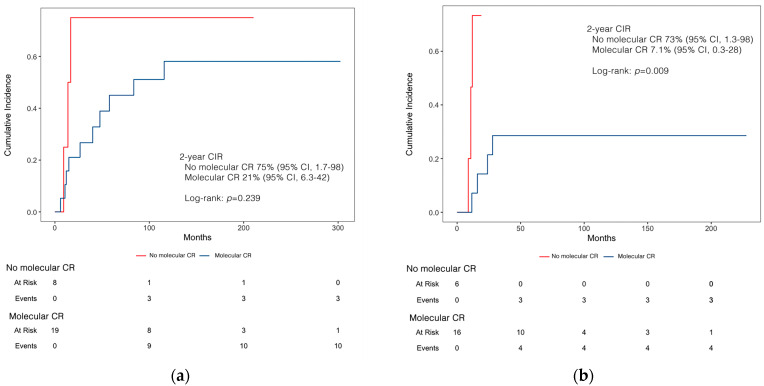
2-year cumulative incidence of relapse (CIR) according to molecular response in (**a**) chemo-based and (**b**) ATO-based treatment cohort.

However, the 2-year CIR of second relapse was 66% (95% CI, 23–89) for patients in molecular CR at the end of salvage therapy, compared to 24% (95% CI, 21–46) in patients positive for the *PML::RARA* transcript (*p* = 0.034). Notably, at a median follow-up time of 37 months (IQR: 11.3–302.4), the 2-year CIR was 75% (95% CI, 1.7–98) in patients without molecular CR who received chemotherapy, significantly higher compared to the 21% (95% CI, 6.3–42) of patients with molecular CR, although not statistically significant (*p* = 0.239) (Figure 3a). Conversely, at a median follow-up time of 27.2 months (IQR: 1.2–7.6) the 2-year CIR was significantly lower in patients treated with ATO ± ATRA who tested negative for the *PML::RARA* transcript, compared to patients without molecular CR [7.1% (95% CI, 0.3–28) vs. 73% (95% CI, 1.3–98), *p* = 0.009] (Figure 3b).

In univariate analysis (Table 5), achieving molecular CR was the only predictor of lower relapse rate [HR 0.355 (95% CI, 0.1–0.9), *p* = 0.035], and this was confirmed in the multivariate analysis [HR 0.338 (95% CI, 0.1–0.9), *p* = 0.036].

*Summary*: HCT did not impact second relapse rates. Notably, patients who were lacking in molecular CR after salvage therapy had a significantly higher 2-year CIR. Molecular CR emerged as the sole predictor of a second relapse.

## 4. Discussion

Despite therapeutic advances, APL relapse remains an unresolved issue in 10% of patients, typically occurring within the first 3 years after completion of first-line therapy [17]. Our analysis revealed a high overall CR rate of 98.2% following salvage therapy, with more than 70% of patients achieving molecular CR. Specifically, all patients treated with ATO-based regimens reached CR, and 70% attained molecular CR, which aligns with the existing literature [18,19]. Notably, most patients receiving ATO-based salvage therapy had previously been treated with chemo-based regimens as first-line therapy. This observation supports the use of ATO-based salvage therapies, consistent with the ELN 2019 guidelines [8]. However, it is noteworthy that no patients in the chemo-based salvage group had received ATO-ATRA as initial therapy, which limits our ability to assess the impact of this treatment approach in our analysis.

We also reported on 29 patients who relapsed during first-line maintenance therapy. To date, maintenance therapy remains the standard for high-risk patients treated with ATRA and chemotherapy [8,16]. Notably, 12 out of 29 patients were classified as high Sanz risk at diagnosis. Indeed, a relapse during first-line maintenance therapy correlated with significantly worse survival compared to those who completed or did not receive maintenance (*p* = 0.045), confirming early relapse as a predictor of poor outcomes.

In our cohort, the 5-year OS rates were similar between transplanted and non-transplanted patients. Taking into account the small sample size, selection biases, and treatment heterogeneity, these results are in contrast with the literature generally indicating inferior outcomes for non-transplanted patients [12,20,21]. This is likely favored by the high rate of non-transplanted patients treated with ATO as salvage therapy in our cohort. For transplanted patients, results appear consistent with a recent meta-analysis that evaluated the impact of autologous HCT compared to allogeneic HCT in the treatment of relapsed APL [22]. As reported by the authors, the use of autologous HCT was associated with an OS of 82% compared to 58% associated with allogeneic transplantation, thus emphasizing the potential advantage of autologous HCT in achieving higher survival rates in relapsed APL patients. It is noteworthy that patients undergoing allogeneic HCT were younger compared to those receiving autologous HCT, which may have influenced different outcomes. This age discrepancy can be attributed to several factors, including the availability of suitable donors and the treatment protocols prevalent at the time [23]. Additionally, four of the patients who underwent allogeneic HCT experienced extramedullary relapses, which could have influenced the clinical decision to prefer allogeneic HCT over autologous HCT, even in older patients.

Sasaki et al. [14] recently reported on 61 relapsed APL patients, comparing pre-ATO-ATRA salvage therapy with modern ATO-ATRA regimen, with or without GO and IDA. They found no difference in CR achievement or OS between the cohorts, irrespective of HCT. These findings suggest that HCT may not be needed after second remission. Our study showed no difference in OS between transplanted and non-transplanted patients treated with ATO-based regimens, while survival advantages were more pronounced in those treated with chemo-based regimens. We observed a significant positive effect of ATO therapy over chemo-based or ATRA ± GO therapy (*p* = 0.035), independent of HCT (Table 4). Achieving molecular CR was associated with reduced second relapse rate in univariate and multivariate analyses, making it the most significant predictor of disease relapse.

Our study has several limitations. First, its retrospective nature may introduce selection bias and unknown confounding factors, affecting the generalizability of the results. Additionally, the study spans a long period (1981–2021), during which treatment protocols and diagnostic technologies evolved significantly, potentially impacting data comparability across different periods. Despite these limitations, we here provide valuable insights into the treatment of relapsed APL, further supporting the efficacy of ATO salvage therapy and suggesting potential management strategies for patients in second CR.

## 5. Conclusions

In conclusion, our findings underscore the effectiveness of ATO-based therapies for first relapse in APL and highlight the importance of considering individual patient factors when deciding on HCT. Caution is necessary for patients relapsing during first-line maintenance or with an early relapse after first-line therapy, who may still require HCT consolidation due to more aggressive disease. Molecular monitoring is paramount in relapsed APL and should guide clinical decision-making. Further studies are needed to evaluate the efficacy of different treatment strategies in this patient setting and to identify the prognostic factors that could guide more personalized therapeutic decisions.

## Figures and Tables

**Table 1:** Recommendations and class/level of evidence for the management of APL relapse according to the 2019 European LeukemiaNet (ELN) guidelines.

Recommendations	Class/Level
For patients with confirmed molecular relapse (in independent labs in two consecutive positive PCR), immediate preemptive therapy is crucial to prevent overt relapse.	IIa/B
Salvage therapy for molecular persistence, molecular relapse, or hematologic relapse should be based on prior first-line treatment and the timing of the first relapse.	IV/C
Patients who relapse after ATRA + CHT should be managed with ATRA + ATO; patients who relapse after ATRA + ATO should be managed with ATRA + CHT.^†^	IV/C
Patients achieving CR2 should ideally undergo intensification with either HCT or CHT, if feasible.	IV/C
Allogeneic HCT is recommended for patients who do not achieve a second molecular remission.	IV/C
Autologous HCT is the preferred option for patients with no detectable MRD in the marrow and an adequate PCR harvest.	IIa/B
For patients ineligible for HCT, options include repeated ATO cycles with or without ATRA and/or CHT.	IV/C
For CNS relapse, induction involves weekly triple ITT with MTX, hydrocortisone, and Ara-C until cerebrospinal fluid blast clearance, followed by 6–10 consolidation ITT treatments. Systemic treatment should adhere to the previously mentioned recommendations.	IV/C

Abbreviations: Ara-C—cytarabine; ATRA—all-*trans* retinoic acid; ATO—arsenic trioxide; CHT— chemotherapy; CR1—first complete response; CR2—second CR; HCT—hematopoietic cell transplant; ITT—intrathecal therapy; MRD—measurable residual disease. ^†^ A potential exception to switching to a different treatment may be considered in case of late relapse (e.g., CR1 > 2 years). Adapted from [8].

**Table 2:** Clinical characteristics of 67 first-relapsed acute promyelocytic leukemia (APL) patients.

Variables	
**At baseline**	
**Males, n (%)**	36 (53.7%)
**Median age at diagnosis, years (IQR)**	45.8 (32.2–58.7)
**Sanz risk score, n (%)**	
Standard risk	45 (67.2)
High risk	22 (32.8)
**Morphology, hypergranular/microgranular; n (%)**	50/17 (74.6/25.4)
** Leukocyte count at diagnosis, ×10^9^/L (IQR)**	2.6 (1.3–15.7)
** Platelet count at diagnosis, ×10^9^/L (IQR)**	27.5 (16.7–49.0)
** First-line regimen, n (%)**	
AIDA0493	23 (34.3)
AIDA2000	38 (56.7)
APL0406	3 (4.5)
AIDA + GO	1 (1.5)
IDA + Ara-C	1 (1.5)
DNR	1 (1.5)
**Maintenance therapy, n (%)**	50 (74.6)
**At relapse**	
**Median age at relapse, years (IQR)**	48.2 (34.9–71.3)
**Relapsed during maintenance therapy, n (%)**	29 (58.0)
**Type of relapse, n (%)**	
Molecular relapse	25 (37.3)
Morphological relapse	42 (62.7)
**Extramedullary relapse, n (%)**	11 (16.4)
**Site of extramedullary relapse**	
Skin	1 (9.1)
Central nervous system	9 (81.8)
Paravertebral mass	1 (9.1)
**Time to relapse from diagnosis, median months (IQR)**	17.3 (12.2–35.3)
Molecular relapse	20.9 (12.9–39.9)
Morphological relapse	31.4 (15.5–48.8)
Extramedullary relapse	27.6 (15.0–50.3)

Abbreviations: Ara-C—cytarabine; DNR—daunorubicin; GO—gemtuzumab ozogamicin; IDA—idarubicin; IQR—interquartile range.

**Table 3:** Patient characteristics and outcomes according to salvage therapies.

Variables	ATO ± ATRA	Chemo-Based	ATRA ± GO	*p*
**Total cohort, n (%)**	27 (40.3)	27 (40.3)	7 (10.4)	
**Median age at relapse, years (IQR)**	50.1 (33.0–60.6)	41.7 (33.0–52.3)	65.9 (54.2–74.9)	0.013 *
**Female gender, n (%)**	11 (40.7)	14 (51.8)	2 (28.5)	0.481
**Morphology, hypergranular/microgranular; n (%)**	20/7 (74.0/26.0)	21/6 (77.7/22.3)	4/3 (57.1/42.9)	0.541
**Year of relapse, range**	2000–2023	1984–2007	1996–2018	<0.001 *
**Sanz risk score at diagnosis, n (%)**				0.010 *
Standard risk	17 (63.0)	16 (59.3)	7 (100)	
High risk	10 (37.0)	11 (40.7)	0 (0)	
**First-line regimen, n (%)**				<0.001 *
AIDA0493	1 (3.7)	17 (62.9)	2 (28.6)	
AIDA2000	25 (92.6)	8 (29.7)	3 (42.8)	
APL0406	1 (3.7)	0	1 (14.3)	
AIDA + GO	0	1 (3.7)	0	
IDA + Ara-C	0	1 (3.7)	0	
DNR	0	0	1 (14.3)	
**Response to salvage therapy, n (%)**				0.112
Morphological CR2	6 (27.2)	8 (29.7)	1 (14.3)	
Molecular CR2	16 (72.7)	19 (70.3)	5 (71.4)	
No response	0	0	1 (14.3)	
**Consolidation at CR2, n (%)**				
Auto-HCT, n (%)	2 (7.4)	14 (51.8)	1 (14.3)	0.565
Allo-HCT, n (%)	3 (11.1)	4 (14.8)	2 (28.6)	
**Second relapse, n (%)**	9 (33.3)	13 (48.1)	4 (57.1)	0.539
**Median time from diagnosis of relapse to 2nd relapse, months (IQR)**	11.6 (5.3–22.4)	9.09 (6.9–21.3)	13.5 (3.6–29.0)	0.236

Abbreviations: Ara-C—cytarabine; CR2—second complete remission; DNR—daunorubicin; GO—gemtuzumab ozogamicin; HCT—hematopoietic stem cell transplant; IDA—idarubicin; IQR—interquartile range.

**Table 4:** Predictive factor for survival in both univariate and multivariate analyses.

Variables	Univariate Analysis			Multivariate Analysis		
	HR	95% CI	*p*	HR	95% CI	*p*
**Female gender**	0.685	0.3–1.2	0.273	1.230	0.9–1.0	0.270
**Age at relapse**	1.020	1.0–1.041	0.054	0.430	0.1–1.2	0.453
**Sanz risk score at diagnosis**	0.374	0.6–1.4	0.393	0.73	0.3–1.5	0.390
**Time of relapse from diagnosis**	**0.968**	**0.94–0.99**	0.016 *	0.992	0.9–1.0	0.623
**Relapse during maintenance**	**1.959**	**1.0–3.8**	0.045 *	**2.012**	**1.1–3.8**	0.049 *
**Chemotherapy**	0.674	0.8–3.4	0.169	1.553	0.7–2.9	0.250
**ATO-therapy**	**0.397**	**0.1–0.8**	**0.025**	**0.423**	**0.1–0.89**	0.026 *
**HCT consolidation**	0.516	0.2–1.1	0.087	0.52	0.24–1.1	0.087

Abbreviations: ATO—arsenic trioxide; HCT—hematopoietic cell transplant.

**Table 5:** Predictive factor for relapse in both univariate and multivariate analyses.

Variables	Univariate Analysis			Multivariate Analysis		
	HR	95% CI	*p*	HR	95% CI	*p*
**Female gender**	1.183	0.5–2.5	0.669	1.348	0.5–3.2	0.509
**Age at relapse**	0.993	0.9–1.0	0.538	0.999	0.9–1.03	0.946
**ATO-therapy**	0.637	0.2–1.4	0.274	0.852	0.3–2.3	0.759
**HCT consolidation**	1.325	0.6–2.9	0.481	1.207	0.3–3.8	0.752
**Molecular CR**	**0.355**	**0.1–0.9**	0.035 *	**0.338**	**0.1–0.9**	0.036 *

Abbreviations: ATO—arsenic trioxide; CR—complete remission; HCT—hematopoietic cell transplant.

## Data Availability

The data that support the findings of this study are available from the corresponding author upon reasonable request.
